# Supplementary material for: Survival, Growth Performance, and Hepatic Antioxidant and Lipid Profiles in Infected Rainbow Trout (Oncorhynchus mykiss) Fed a Diet Supplemented with Dihydroquercetin and Arabinogalactan
Source: Animals (Basel). 2023 Apr 14;13(8):1345. doi: 10.3390/ani13081345 (PMC10135201; doi:10.3390/ani13081345)
Supplement: Supplementary file 1 [file animals-13-01345-s001.zip › animals-2263449-supplementary.pdf]

Table S1. Chemical composition of Efico alpha 717 diets according to the producer's specification (BioMar, Denmark).

Content	Pellet fraction 3.0 mm	Pellet fraction 4.5 mm
Crude protein, %	39-42	39-42
Lipids, %	21-24	21-24
Carbohydrates, %	19-22	19-22
Alimentary fiber, %	4-5.9	4-5.9
Ash substances, %	4-7	4-7
Total phosphorus, %	0.8	0.8
Energy for growth, mJ/kg	21-24	21-24
Digestible energy, mJ/kg	18.3	18.3

Table S2. Lipid (% dry weight) and fatty acid (wt% total fatty acids) composition of Efico alpha 717 feed pellets.

**Size of pellets**	**3.0 mm**	**4.5 mm**
Lipid content		
Total lipids	23.28±0.37	24.03±0.53
Phospholipids	0.00±0.00	0.16±0.24
Triacylglycerols	18.68±0.45	18.55±0.94
Cholesterol esters	0.00±0.00	0.00±0.00
Cholesterol	4.60±0.40	5.32±0.63
Fatty acids		
12:00	0.05±0.01	0.04±0.02
14:00	2.23±0.08	1.73±1.11
14:01	0.09±0.01	0.10±0.02
15:00	0.18±0.01	0.14±0.08
15:01	0.03±0.01	0.08±0.06
16:0	9.41±0.38	7.25±4.70
16:1n-7	2.27±0.09	1.70±1.04
17:00	0.31±0.04	0.29±0.16
17:01	0.21±0.05	0.18±0.11
18:0	2.54±0.25	2.00±1.15
18:1n-7	3.50±0.46	2.65±1.47
18:1n-9cis	43.03±0.43	32.39±21.03
18:2n-6cis	16.04±0.13	11.99±7.70
18:3n-6	0.19±0.13	0.42±0.35
18:3n-3	7.66±0.15	5.71±3.80
20:00	0.39±0.03	0.32±0.19
20:1n-9	3.34±0.62	2.43±1.70
20:4n-6	0.18±0.04	0.15±0.09
20:3n-3	0.10±0.01	0.10±0.02
20:3n-6	0.05±0.02	0.08±0.02
20:5n-3	3.54±0.17	2.73±1.73
21:00	0.15±0.03	0.13±0.04
22:00	0.10±0.03	0.09±0.02
22:1n-9	0.20±0.10	0.21±0.08
22:2n-6	0.08±0.01	0.08±0.03
22:6n-3	3.44±0.50	2.61±1.67
23:00	0.18±0.01	0.18±0.06
24:00	0.31±0.11	0.24±0.14
24:1n-9	0.14±0.04	0.18±0.04
n-3/n-6	1.05±0.01	1.1±0.01

Table S3. Water quality at various distance from fish farming cages:
1 – 500 m north of the cages, 1 m depth; 2 – 500 m north of the cages, 5 m depth; 3 – in cages, 1 m depth; 4 – in cages, 5 m depth; 5 – 500 m south of cages, 1 m depth; 6 – 500 m south of cages, 5m depth .

**Sampling date**	**29.06**						**04.10**					
**Indicators**	**1**	**2**	**3**	**4**	**5**	**6**	**1**	**2**	**3**	**4**	**5**	**6**
pH, units pH	6.69±0.05	6.69±0.05	6.94±0.05	6.82±0.05	6.66±0.05	6.67±0.05	7.18±0.05	7.30±0.05	7.15±0.05	6.92±0.05	7.22±0.05	6.85±0.05
Suspended substances, mg/ml	6.2±1.1	5.9±1.1	6.0±1.1	6.1±1.1	5.4±1.1	6.0±1.1	0.7±0.1	0.9±0.2	0.8±0.2	1.1±0.2	0.9±0.2	1.0±0.2
BOD 5, mgO_2/ml	0.8±0.2	0.9±0.2	0.7±0.2	0.8±0.2	0.9±0.2	0.6±0.2	0.7±0.2	0.8±0.2	0.7±0.2	0.5±0.2	0.7±0.2	0.8±0.2
$PO4^{3-}$, mg/ml	< 0.25	< 0.25	< 0.25	< 0.25	< 0.25	< 0.25	< 0.25	< 0.25	< 0.25	< 0.25	< 0.25	< 0.25
Phosphorus, mg/ml	< 0.02	< 0.02	< 0.02	< 0.02	< 0.02	< 0.02	< 0.02	< 0.02	< 0.02	< 0.02	< 0.02	< 0.02
$NH4^{+}$, mg/ml	< 0.5	< 0.5	< 0.5	< 0.5	< 0.5	< 0.5	< 0.5	< 0.5	< 0.5	< 0.5	< 0.5	< 0.5
Oil products, mg/ml	0.007±0.004	0.007±0.004	0.006±0.003	< 0.005	0.005±0.003	< 0.005	0.0075±0.0037	< 0.005	0.0098±0.0049	< 0.005	< 0.005	0.008±0.004
Iron, mg/ml	0.012±0.003	0.0019±0.0004	0.0015±0.0004	0.0017±0.0004	0.0022±0.0004	0.0014±0.0004	< 0.001	< 0.001	< 0.001	< 0.001	< 0.001	< 0.001
Permanganate oxidation, mg O_2/ml	4.6±0.5	4.7±0.5	4.6±0.5	5.1±0.5	5.0±0.5	4.8±0.5	3.8±0.4	3.9±0.4	3.7±0.4	4.0±0.4	3.8±0.4	4.0±0.4

Table S4. Fish mortality (cumulative deaths, % of all fish) over the observation period.

Date	Supplement-free	Supplement-fed
06.07	0.0	0.0
31.07	0.9	0.9
31.08	3.1	1.6
30.09	4.3	2.2
31.10	4.4	2.3
30.11	6.5	2.5

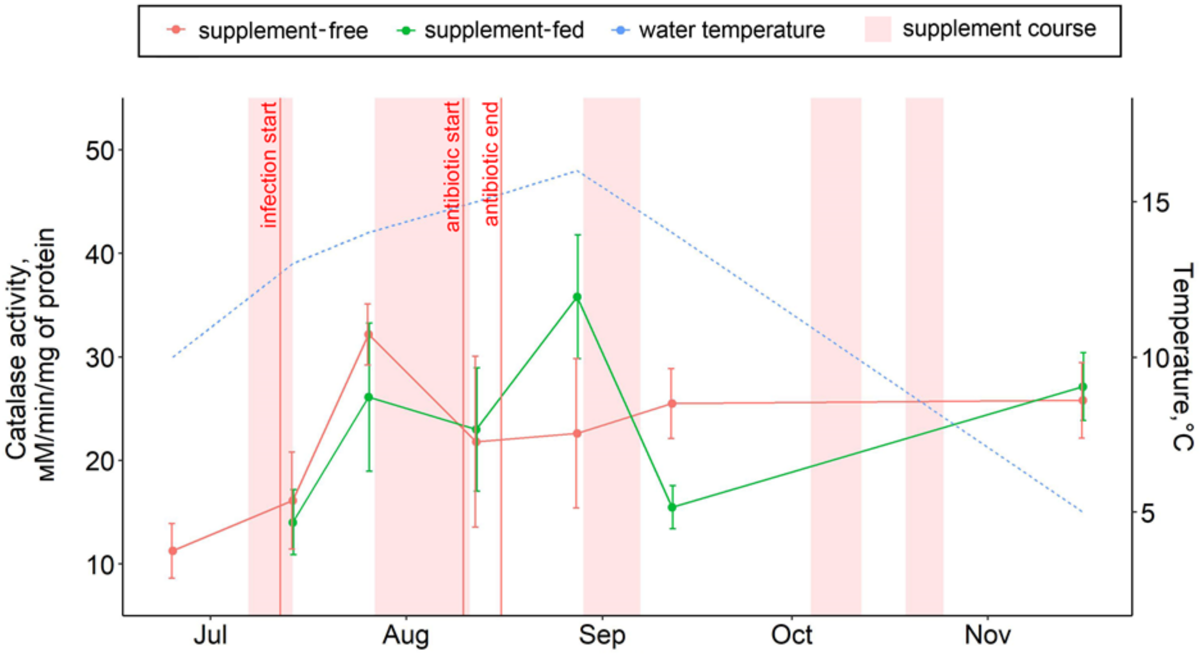

Figure S1. Hepatic catalase activity in supplement-free and supplement-fed *O. mykiss*.

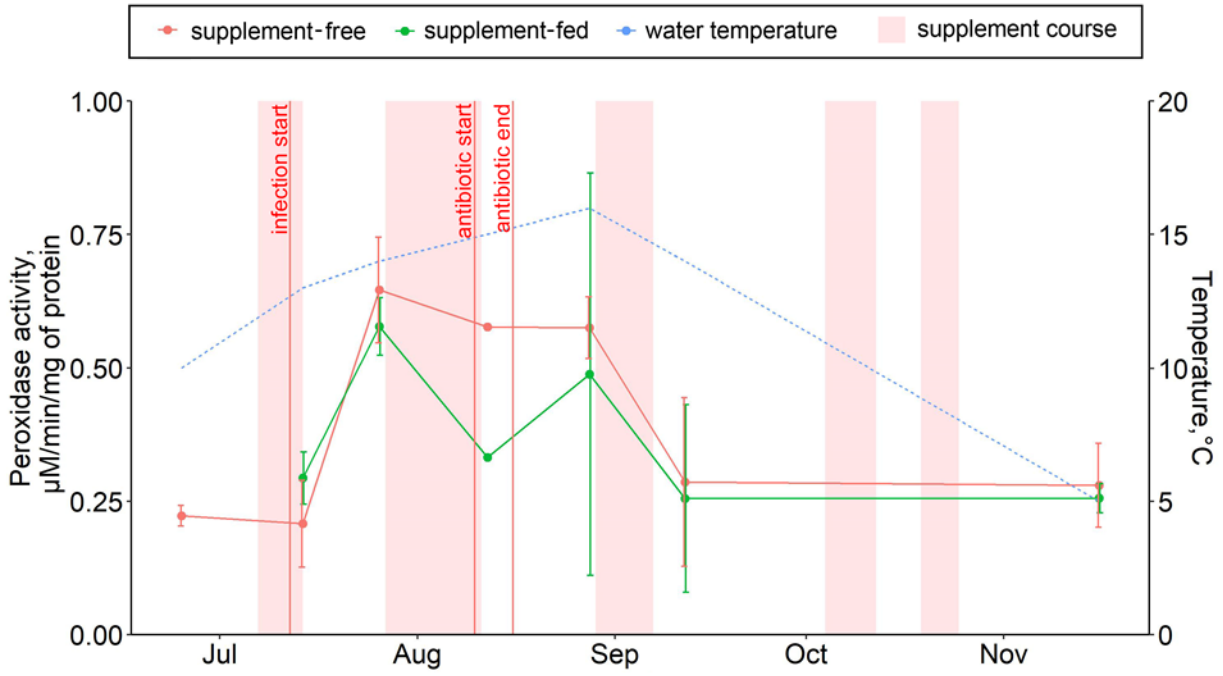

Figure S2. Hepatic peroxidase activity in supplement-free and supplement-fed *O. mykiss*.

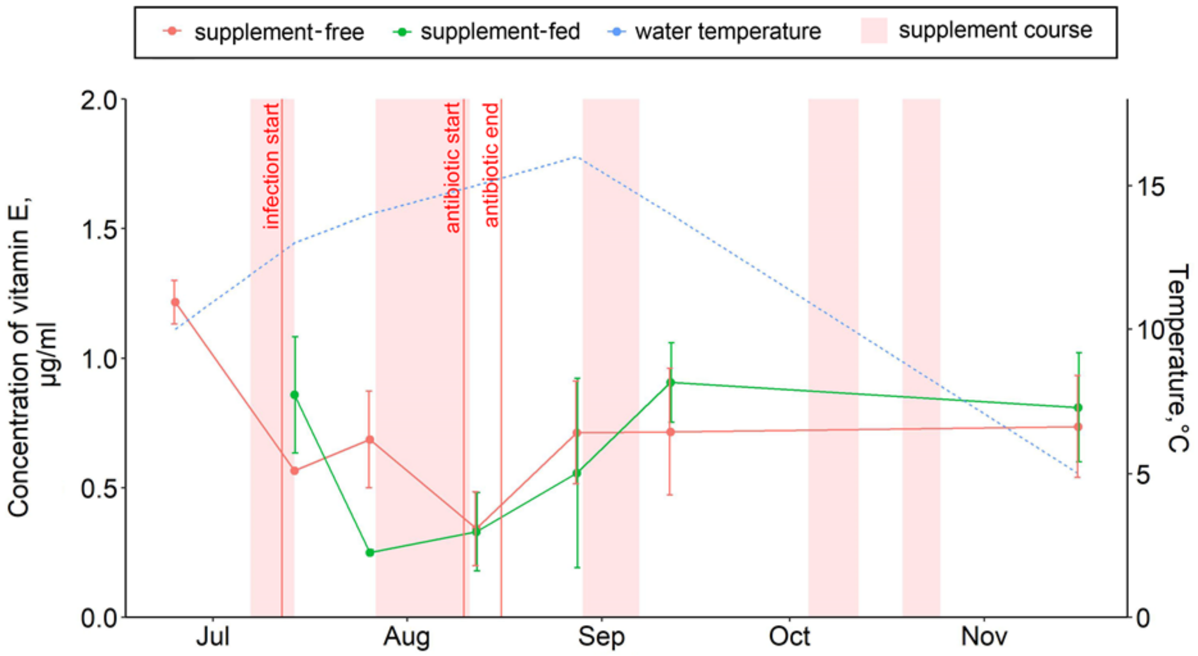

Figure S3. Hepatic vitamin E content in supplement-free and supplement-fed *O. mykiss*.

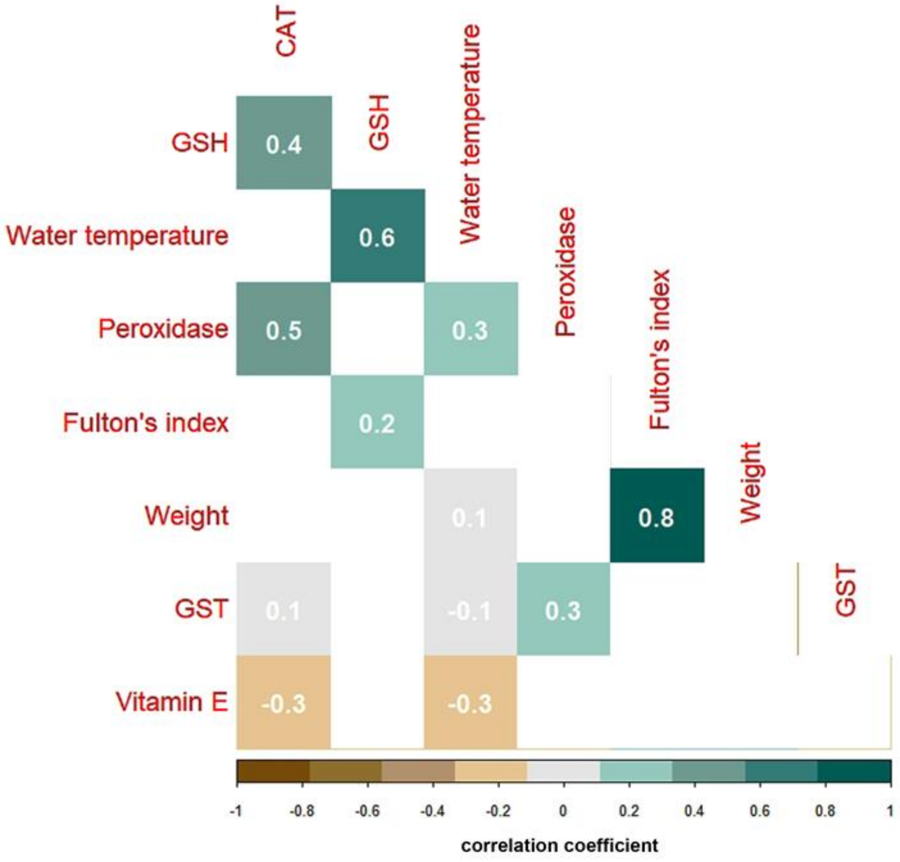

Figure S4. Correlations of antioxidant components in *O. mykiss* liver with fish weight and water temperature (p≥0.05 not shown). Abbreviations: CAT, catalase; GSH, reduced glutathionet; GST, glutathione S-transferase .

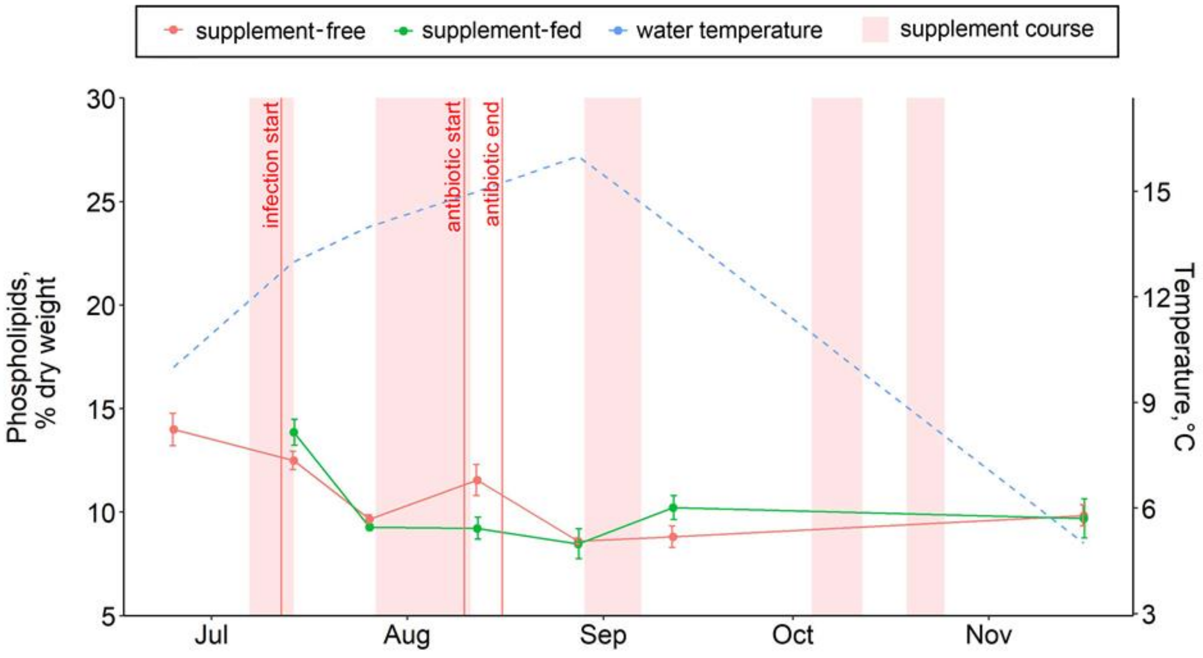

.

Figure S5. Hepatic phospholipid content in supplement-free and supplement-fed *O. mykiss.*

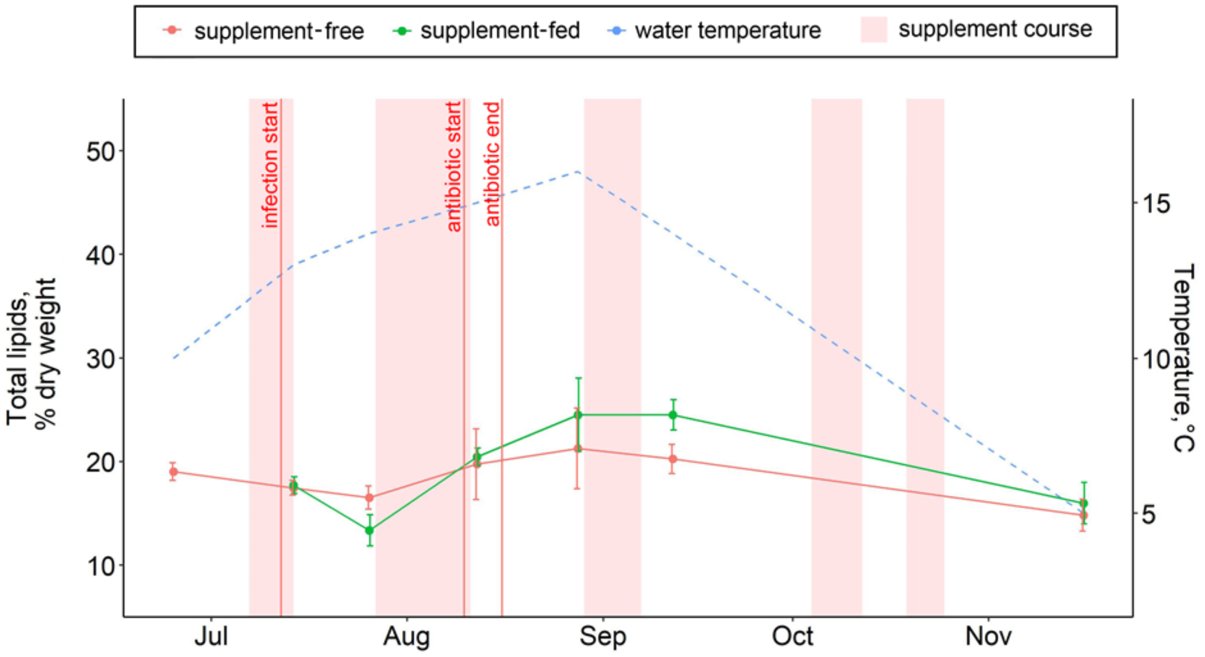

Figure S6. Hepatic total lipid content in supplement-free and supplement-fed *O. mykiss*.

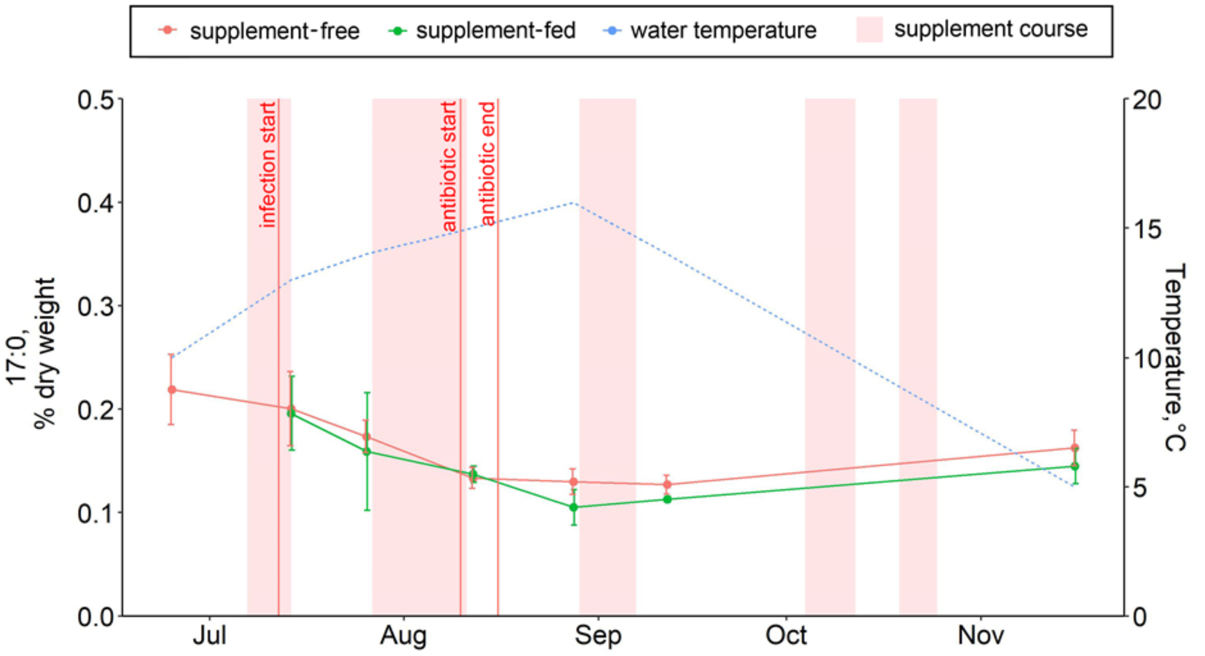

Figure S7. Hepatic 17:0 fatty acid content in supplement-free and supplement-fed *O. mykiss*.

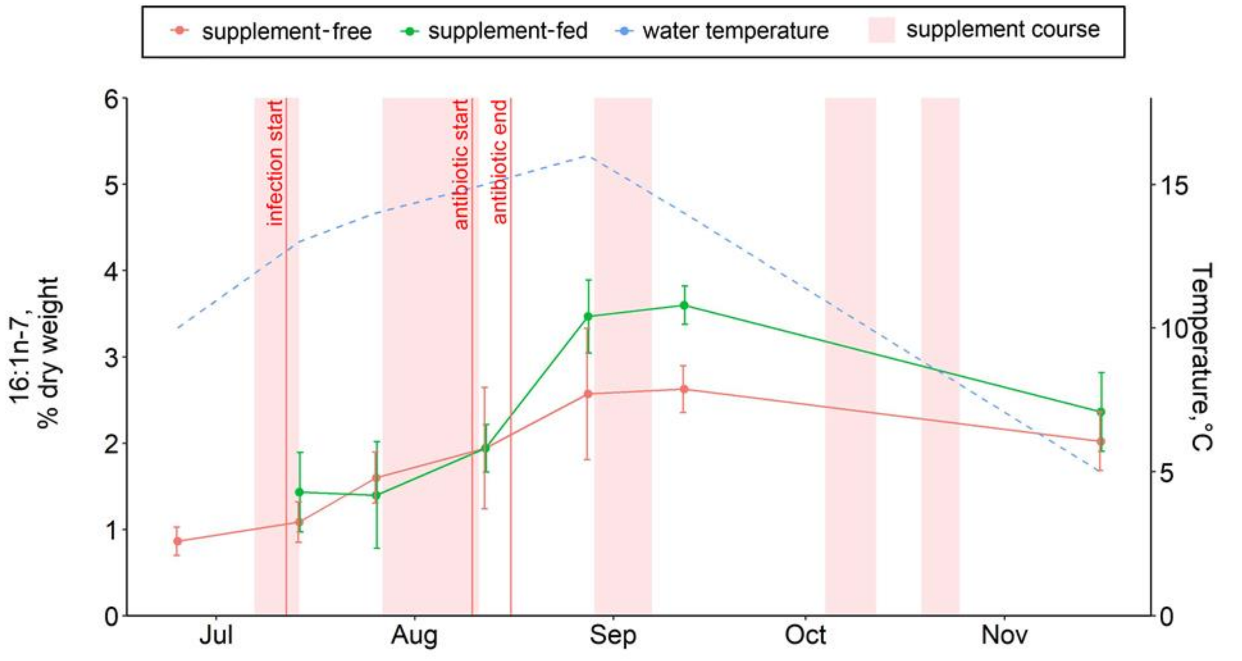

Figure S8. Hepatic palmitoleic 16:1n-7 fatty acid content in supplement-free and supplement-fed *O. mykiss*.

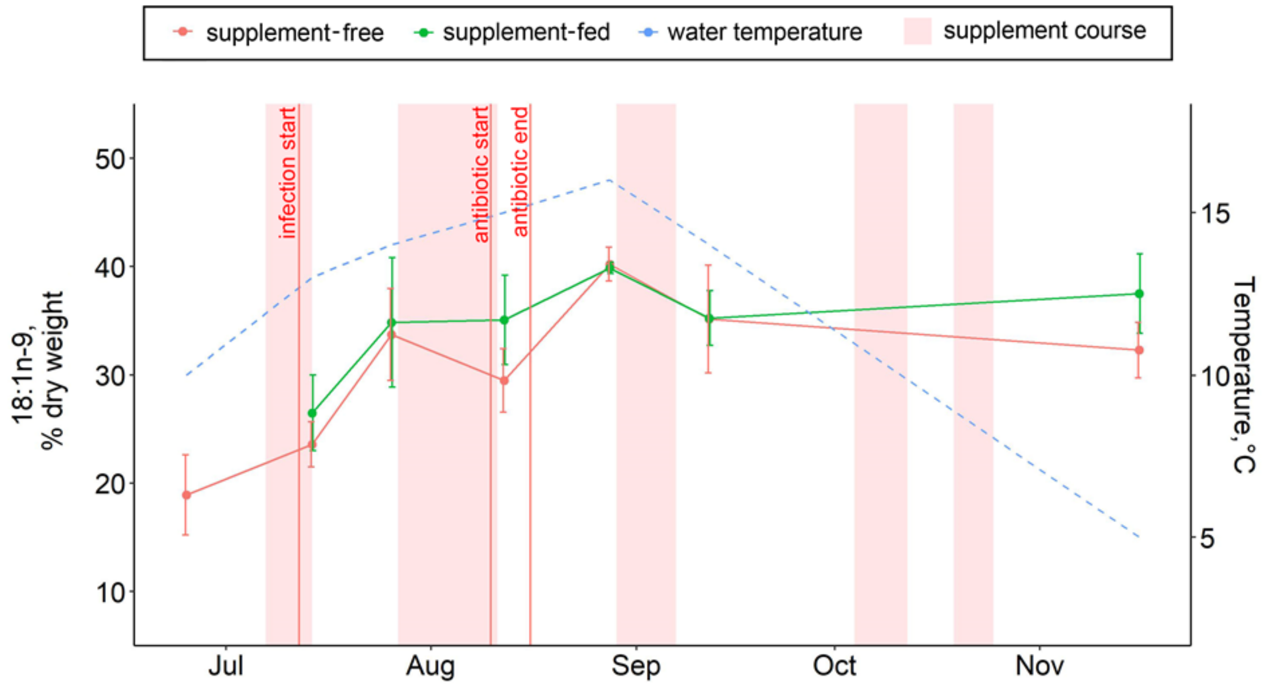

Figure S9. Hepatic oleic 18:1n-9 fatty acid content in supplement-free and supplement-fed *O. mykiss*.

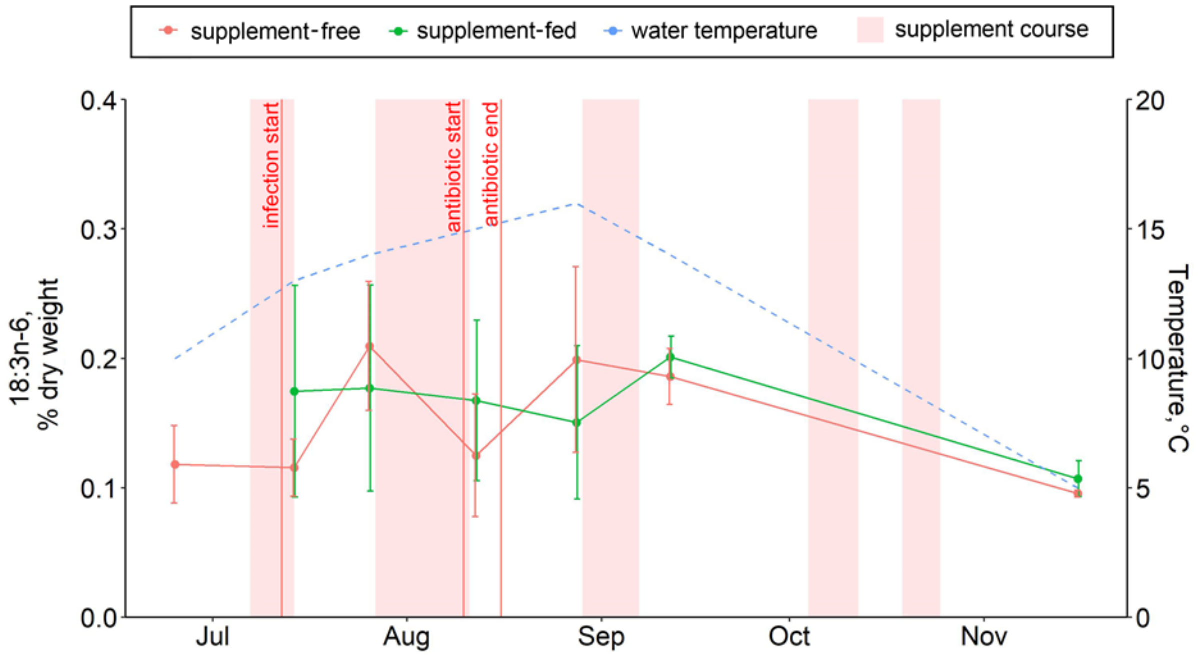

Figure S10. Hepatic gamma-linolenic 18:3n-6 fatty acid content in supplement-free and supplement-fed *O. mykiss*.

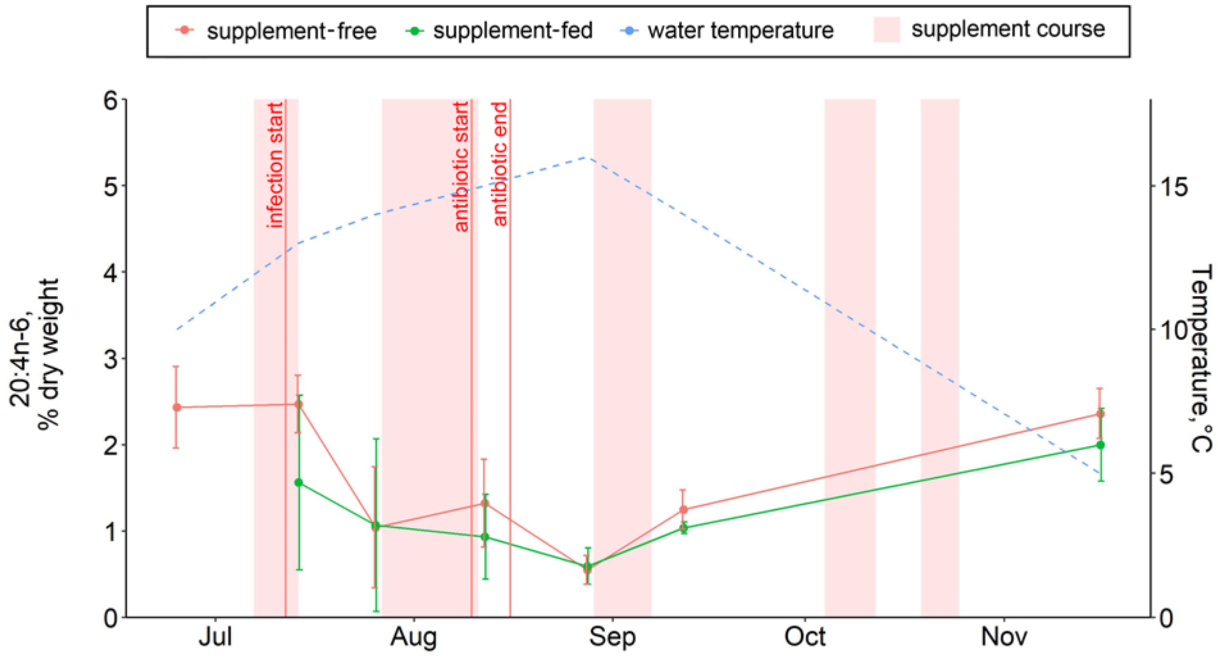

Figure S11. Hepatic arachidonic 20:4n-6 fatty acid content in supplement-free and supplement-fed *O. mykiss*.

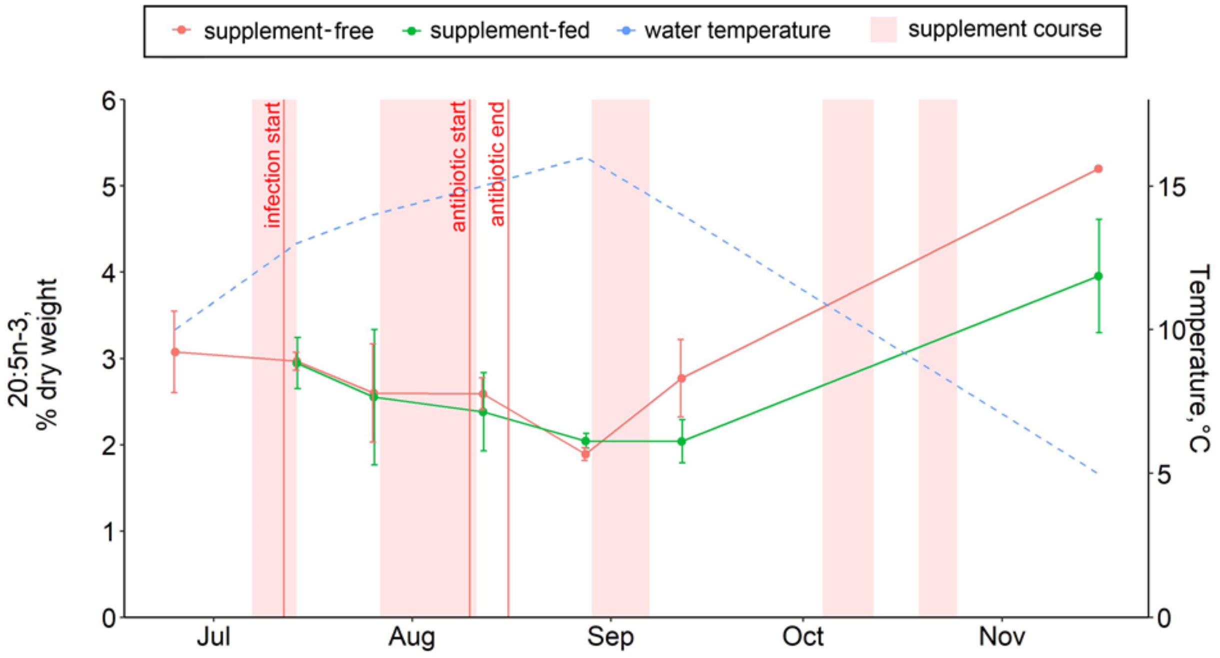

Figure S12. Hepatic eicosapentaenoic 20:5n-3 fatty acid content in supplement-free and supplement-fed *O. mykiss*.

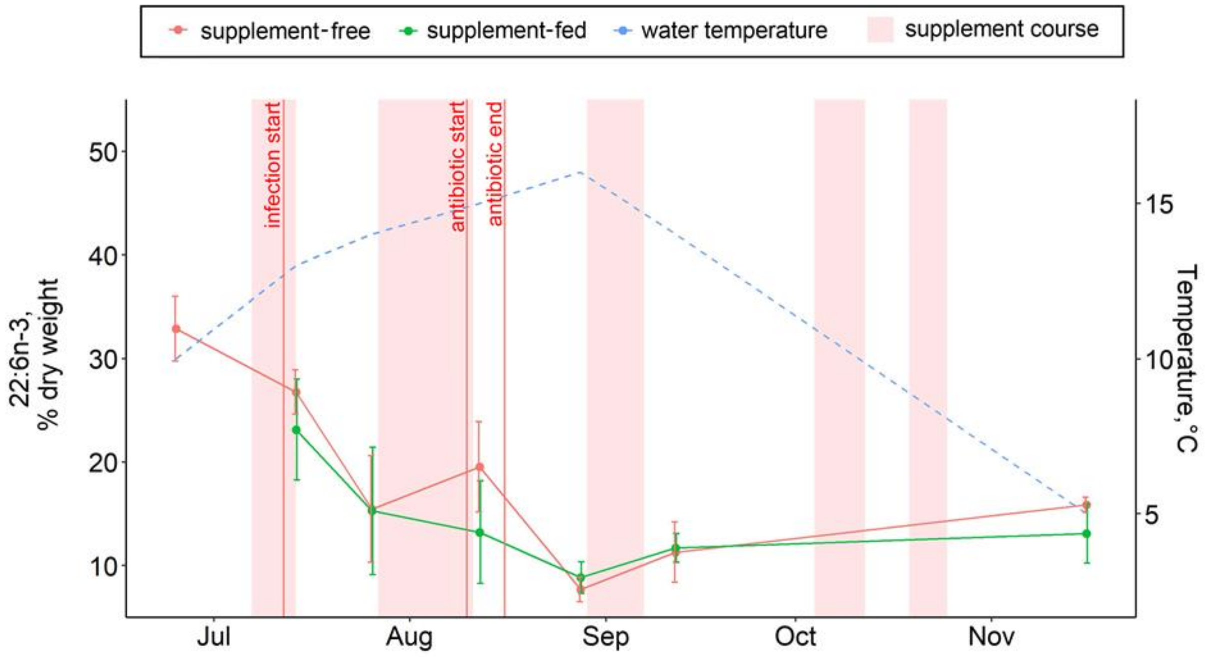

Figure S13. Hepatic docosahexaenoic 22:6n-3 fatty acid content in supplement-free and supplement-fed *O. mykiss*.
